# Disparities in untreated caries among children and adults in the U.S., 2011–2014

**DOI:** 10.1186/s12903-018-0493-7

**Published:** 2018-03-06

**Authors:** Niodita Gupta, Marko Vujicic, Cassandra Yarbrough, Brittany Harrison

**Affiliations:** American Dental Association, Health Policy Institute, 211 E. Chicago Ave., Chicago, IL 60611 USA

**Keywords:** National Health and Nutrition Examination Survey, Untreated caries, Oral health disparities, Financial and non-financial barriers

## Abstract

**Background:**

The Affordable Care Act of 2010 increased dental coverage for children in the United States, (U.S.) but not for adults. Few studies in current scholarship make use of up-to-date, nationally representative data to examine oral health disparities in the U.S. population. The purpose of this study is to use nationally representative data to determine the prevalence of untreated caries among children and adults of different socioeconomic and racial/ethnic groups and to examine the factors associated with untreated caries among children and adults.

**Methods:**

This study used the 2011–2014 National Health and Nutrition Examination Survey (NHANES) demographic, oral health questionnaire, and oral health dentition examination data (*n* = 7008 for children; *n* = 9673 for adults). Participants that had a standardized oral health examination and at least one natural primary or permanent tooth considering 28 tooth spaces were included in this study. Our main outcome measure was untreated coronal caries defined as decay on the crown or enamel surface of a tooth that had not been treated or filled. Population estimates were calculated to determine the prevalence of untreated caries among children and adults in the United States. Frequencies and Pearson’s chi-square tests were used to compare those with and without untreated caries. Multivariate logistic regression models were used to evaluate the factors associated with untreated caries. We conducted analyses among children and adults separately.

**Results:**

From 2011 to 2014, 12.4 million children and 57.6 million adults in the United States had untreated caries. Age, family income level, recent dental visit, and financial and non-financial barriers were significantly associated with untreated caries in both children and adults. Race/ethnicity, gender and education level were also significantly associated with untreated caries among adults. The odds of untreated caries associated with financial barriers were 2.06 for children and 2.84 for adults while the odds of untreated caries associated with non-financial barriers were 2.86 for children and 1.67 for adults.

**Conclusions:**

Demographic and socio-economic disparities in untreated caries exist among children and adults.

## Background

Evidence continues to suggest that oral health is linked to overall health, and dental care utilization may lead to health care cost savings among children and adults [1, 2]. Yet oral health is still regarded as optional in health policy for most of the United States population. Oral health services are considered essential for children under the Affordable Care Act (ACA), and Medicaid and the Children’s Health Insurance Program (CHIP) have been required to cover dental benefits for children since 2010 [3]^.^ Expanded coverage has led to increased dental care utilization among children under 19 years of age [4]. Despite this progress, about 11% of children forgo dental benefits coverage [5] and about 5% are unable to access dental services due to cost. [6] Furthermore, there are no coverage requirements for working-age adults or seniors, which is reflected in downward trends in dental benefits coverage [5] and dental care utilization among adults [4].

Globally, dental caries are a common chronic condition among people of all ages [7]. Untreated caries can lead to significant pain and infection that require extensive treatment [7]. Previous studies have identified various factors associated with the prevalence of untreated caries such as race, [8–11] socio-economic status, [7, 11–14] rural area residence, [15] insurance type, [15] and age category [14, 15]. A recent study suggests that the prevalence of untreated caries among young children has lowered in the U.S. since the implementation of the ACA [16]. However, there is a gap in the literature on disparities in untreated caries in the U.S. post-ACA. Most studies examine disparities using data prior to implementation of the ACA or fail to use a nationally representative sample. To the best of our knowledge, no study has examined the association of utilization of care and barriers to accessing dental care with untreated caries post-ACA. Examining post-ACA nationally representative data for both children and adults could shed light on potential persistent disparities and factors associated with untreated caries. In this paper, we aimed to determine the prevalence of untreated caries in the U.S. population. We examined factors associated with untreated caries among children and adults separately. We hypothesized that improved access to and utilization of dental care post-ACA are associated with lower odds of untreated caries among children and adults.

## Methods

The National Health and Nutrition Examination Survey (NHANES) is conducted annually by the National Center for Health Statistics at the Centers for Disease Control and Prevention [17]. NHANES uses a stratified, multistage sampling design to survey the civilian non-institutionalized U.S. population across 50 states and the District of Columbia [17]. NHANES data are considered nationally representative. The survey includes interviews conducted at home and standardized health examinations conducted in specially equipped mobile examination centers. We used the demographic, oral health questionnaire, and oral health dentition examination data from NHANES for the years 2011 through 2014. Because the questionnaires and the method of oral examination were the same for the survey cycles 2011–2012 and 2013–2014, these data were considered comparable and were merged together. During 2011–2014, oral examinations were conducted by trained and licensed dentists on all eligible survey participants ages one and older. To obtain population counts of the civilian non-institutionalized U.S. population by gender, age, and race/ethnicity, annual microdata sample files were used from the American Community Survey for the years 2011 and 2013 [18].

We only included survey respondents that had a standardized oral health examination and at least one natural primary or permanent tooth considering 28 tooth spaces. We conducted analyses separately for children and adults. Children were defined as individuals under 19 years of age and adults as 19 years of age and above. The study sample included 7008 children and 9673 adults.

### Dependent variable: untreated caries

Untreated coronal caries, our main outcome measure, refers to decay on the crown or enamel surface of a tooth that has not been treated or filled. This was a binary variable categorized as “yes” or “no.” According to NHANES examination methodology, a dental lesion is considered to be untreated only if it is cavitated lesion [19]. Thus, non-cavitated lesions and decay in the root were not included in this study. The third molars were also not included in the calculation. Untreated caries were evaluated in both primary and permanent dentition. Untreated caries in mixed dentition were calculated by combining untreated caries in primary and/or permanent dentition.

### Independent variables: demographic and socioeconomic characteristics

Age is a continuous variable in NHANES data which was categorized as 0 to 5 years, 6 to 11 years, and 12 to 18 years for children and 19 to 45 years, 46 to 64 years, and 65 years and above for adults. Race/ethnicity was categorized as Mexican American, other Hispanic, non-Hispanic White, non-Hispanic Black, and other race (including multi-racial) for both children and adults. This categorization is based on the NHANES categorization of race and Hispanic origin. Gender was categorized as male and female. The federal poverty level (FPL) is used to determine an individual’s eligibility to receive benefits for programs such as Medicaid or CHIP. In 2017, the 100% FPL was $12,060 for an individual or $16,240 for a family of two [20]. Ratio of family income to poverty is a continuous variable in NHANES and was used to calculate family income categories. Family income categories based on FPL were categorized as less than 100% FPL, 100–199% FPL, 200–399% FPL, and 400% FPL and above. Marital status was categorized as “with a significant other” if the respondent indicated being married or living with a partner and as “without a significant other” if the respondent indicated being widowed, divorced, separated or never married. Education level was categorized as “not a high school graduate,” “high school graduate/GED,” and “some college and above.”

Access to dental care was measured in terms of barriers to obtaining dental care when one needed a dental visit. The NHANES question, “What were the reasons that (you/SP [sample person]) could not get the dental care (you/she/he) needed?” was used to determine barriers. NHANES data has a separate variable for each reason. In order to combine the reasons into financial and non-financial, we created dummy variables for each reason. Each dummy variable was coded as 1 if a reason was selected and 0 if that reason was not selected If any of the following reasons were coded as 1 for a participant, then they were categorized as “yes” for financial barriers: could not afford the cost, did not want to spend the money, or insurance did not cover procedures. If all three of these reasons were coded as 0, then the participant was categorized as “no” for financial barriers. If any of the following reasons were coded as 1 for a participant, then they were categorized as “yes” for non-financial barriers: dental office is too far away, office not open at convenient time, another dentist recommended not doing it, afraid or do not like dentists, unable to take time off from work, too busy, expected dental problems to go away, or other reason. If all eight of these reasons were coded as 0, then the participant was categorized as “no” for non-financial barriers. The utilization of dental care services was measured by using the NHANES question, “About how long has it been since (you/SP) last visited a dentist? Include all types of dentists, such as, orthodontists, oral surgeons, and all other dental specialists, as well as dental hygienists.” This is a categorical variable in NHANES with multiple time periods. We re-categorized the recent dental visit variable as “less than one year” and “more than one year.”

### Data analysis

Descriptive statistics such as weighted frequencies and percentages of children and adults with untreated caries were calculated to account for the complex sampling nature of the NHANES survey. Using these estimates and the population counts from the American Community Survey data, the number of children and adults in the U.S. with untreated caries was calculated. Pearson chi-square tests were performed for each independent variable. Based on the results of the Pearson chi-square tests, independent variables were included in multivariate logistic regression models. Multivariate logistic regression models were conducted for children and adults separately to examine the factors associated with untreated caries. The difference in the percentages of children with and without untreated caries was significantly different for all characteristics except gender. Hence, gender was not included in the multivariate logistic regression model. For children, the independent variables included were age, race/ethnicity, family income, recent dental visit, and financial and non-financial barriers. The difference in the percentage of adults with and without untreated caries was significantly different for each characteristic. Hence, all of these independent variables were included in the multivariate logistic regression model. For adults, the independent variables included were age, race/ethnicity, gender, family income, marital status, education level, recent dental visit, and financial and non-financial barriers. The F-adjusted mean residual test was conducted using svylogitgof in STATA to assess the goodness of fit of the models. [21] Data manipulation and population estimates were calculated using SAS 9.4 software and descriptive statistics, bivariate analyses, and regression analyses were conducted using STATA 14.0.

## Results

The prevalence of untreated caries in the U.S. during 2011–2014 was 15.9% (12.4 million) for children and 25.0% (57.6 million) for adults (see Table 1).

**Table 1:** Estimated number and percent of children and adults in the U.S. with untreated caries, 2011–2014

Characteristics	Number of children in the U.S. with untreated caries	Percent of children in the U.S. with untreated caries	Number of adults in the U.S. with untreated caries	Percent of adults in the U.S. with untreated caries
Untreated caries	12,421,000	15.90	57,648,000	24.98
Age				
0 to 5 years	2,193,000	9.14	–	–
6 to 11 years	4,583,000	18.61	–	–
12 to 18 years	5,394,000	18.28	–	–
19 to 45 years	–	–	32,576,000	29.28
46 to 64 years	–	–	17,302,000	22.24
65 years and above	–	–	6,951,000	16.66
Gender				
Male	6,661,000	16.68	30,134,000	27.17
Female	5,759,000	15.08	27,438,000	22.89
Race/Ethnicity				
Mexican American	2,740,000	21.3	7,700,000	37.09
Other Hispanic	886,000	15.73	3,625,000	27.92
Non-Hispanic White	5,694,000	13.81	31,956,000	20.85
Non-Hispanic Black	2,170,000	20.13	10,485,000	39.58
Other race, including multi-racial	941,000	12.35	3,983,000	23.06

During 2011–2014, the total U.S. child population was 78,123,000 and the total U.S. adult population was 230,775,000

Mexican American children had the highest percentage of untreated caries at 21.3%. About 21.7% of children under 100% FPL had untreated caries whereas only 8.0% of children at 400% FPL and above had untreated caries. Among children who faced financial barriers to receiving dental care when needed, about 39.0% had untreated caries. About 47.8% of the children who faced non-financial barriers to receiving dental care when needed had untreated caries. For full descriptive statistics and multivariate logistic regression model results, see Table 2.

**Table 2:** Frequency, percentage and results of multivariate logistic regression for untreated caries among children in the U.S., 2011–2014

Characteristic	With Untreated caries n (weighted percent)	Without untreated caries n (weighted percent)	*p*-value^a^	Odds Ratio^b^ (95% CI) for untreated caries
Age				
0 to 5 years	239 (9.14)	1991 (90.86)	< 0.001	0.38 (0.29–0.51)**
6 to 11 years	518 (18.61)	2005 (81.39)		1.16 (0.92–1.47)
12 to 18 years	436 (18.28)	1819 (81.72)		1 (ref)
Race/Ethnicity				
Mexican American	294 (21.30)	1159 (78.70)	< 0.001	1.18 (0.90–1.54)
Other Hispanic	128 (15.73)	651 (84.27)		0.89 (0.62–1.27)
Non-Hispanic White	238 (13.81)	1441 (86.19)		1 (ref)
Non-Hispanic Black	376 (20.13)	1545 (79.87)		1.21 (0.97–1.51)
Other race, including multi-racial	157 (12.35)	1019 (87.65)		0.81 (0.59–1.11)
Gender				
Male	629 (16.68)	2960 (83.32)	0.19	–
Female	564 (15.08)	2855 (84.92)		–
Family income categories based on FPL^c^				
Less than 100% FPL	506 (21.73)	1871 (78.27)	< 0.001	2.68 (2.03–3.55)**
100–199% FPL	316 (18.77)	1388 (81.23)		2.25 (1.59–3.19)**
200–399% FPL	187 (12.96)	1189 (87.04)		1.49 (1.04–2.12)*
400% FPL and above	83 (8.03)	956 (91.97)		1 (ref)
Recent dental visit^c^				
Less than one year	794 (13.58)	4369 (86.42)	< 0.001	0.44 (0.35–0.56)**
More than one year	393 (23.26)	1431 (76.74)		1 (ref)
Financial barriers				
Yes	99 (38.98)	155 (61.02)	< 0.001	2.06 (1.42–2.99)**
No	1094 (15.11)	5660 (84.89)		1 (ref)
Non-financial barriers				
Yes	49 (47.80)	42 (52.20)	< 0.001	2.86 (1.24–6.58)*
No	1144 (15.55)	5773 (84.45)		1 (ref)

The sample size was N = 7008; *CI* = Confidence interval

** *p*-value < 0.001; * *p*-value < 0.05

^a^*p*-value from Pearson chi-square statistics comparing the percentage of children with untreated caries and without untreated caries

^b^Odds ratios were determined by multivariate logistic regression model. The F-adjusted mean residual test for goodness of fit suggests no evidence of lack of fit (*p*-value = 0.099). Gender was not included in the multivariate logistic regression model because the *p*-value of gender for Pearson chi-square test was not significant

^c^Family income categories based on FPL had 512 missing observations and recent dental visit had 21 missing observations

The odds of untreated caries among children ages 0 to 5 years were significantly lower than the odds of untreated caries among children ages 12 to 18 years (OR: 0.38; 95% CI: 0.29–0.51). The odds of untreated caries were higher among children from low family income categories. The odds of untreated caries among children below 100% FPL were 2.7 as compared to children at 400% FPL and above (OR: 2.68; 95% CI: 2.03–3.55). A dental visit within the past year was associated with lower odds of untreated caries among children (OR: 0.44; 95% CI: 0.35–0.56). The odds of untreated caries among children with financial barriers were 2.1 (OR: 2.06; 95% CI: 1.42–2.99) while the odds of untreated caries among children with non-financial barriers were 2.9 (OR: 2.86; 95% CI: 1.24–6.58). Demographic factors such as age, socio-economic factors such as family income level, access to care, and utilization of care were significantly associated with untreated caries among children.

Non-Hispanic Black adults had the highest percentage of untreated caries at 39.6%. About 43.2% of adults below 100% FPL had untreated caries. About 44.2% of adults who were not high school graduates had untreated caries. Among the adults who had a recent dental visit in the last year, about 15.7% had untreated caries compared to 41.1% who did not have a dental visit in more than a year. About 54.4% of adults with financial barriers to receiving dental care had untreated caries and 49.9% of adults with non-financial barriers to receiving dental care had untreated caries. The results of these descriptive statistics and the multivariate logistic regression model are summarized in Table 3.

**Table 3:** Frequency, percentage and results of multivariate logistic regression for untreated caries among adults in the U.S., 2011–2014

Characteristic	With Untreated caries n (weighted percent)	Without untreated caries n (weighted percent)	*p*-value^a^	Odds Ratio^b^ (95% CI) for untreated caries
Age				
19 to 45 years	1594 (29.28)	3276 (70.72)	< 0.001	1.47 (1.13–1.91)*
46 to 64 years	874 (22.24)	2162 (77.76)		1.24 (1.02–1.51)*
65 years and above	390 (16.66)	1377 (83.34)		1 (ref)
Race/Ethnicity				
Mexican American	439 (37.09)	738 (62.91)	< 0.001	0.96 (0.77–1.21)
Other Hispanic	233 (27.92)	678 (72.08)		0.72 (0.54–0.95)*
Non-Hispanic White	992 (20.85)	2820 (79.15)		1 (ref)
Non-Hispanic Black	875 (39.58)	1382 (60.42)		1.61 (1.34–1.95)**
Other race, including multi-racial	319 (23.06)	1197 (76.94)		1.01 (0.81–1.26)
Gender				
Male	1511 (27.17)	3213 (72.83)	< 0.05	1.28 (1.06–1.54)*
Female	1347 (22.89)	3602 (77.11)		1 (ref)
Family income categories based on FPL^c^				
Less than 100% FPL	916 (43.23)	1169 (56.77)	< 0.001	2.40 (1.89–3.05)**
100–199% FPL	835 (35.57)	1401 (64.43)		2.00 (1.69–2.36)**
200–399% FPL	560 (23.22)	1692 (76.78)		1.58 (1.29–1.94)**
400% FPL and above	320 (11.42)	2034 (88.58)		1 (ref)
Marital status^c^				
With a significant other	1472 (22.03)	4031 (77.97)	< 0.001	0.88 (0.76–1.02)
Without a significant other	1314 (30.13)	2555 (69.87)		1 (ref)
Education level^c^				
Not a high school graduate	851 (44.19)	1096 (55.81)	< 0.001	1 (ref)
High school graduate/GED	754 (33.50)	1334 (66.50)		0.80 (0.66–0.97)*
Some college and above	1246 (18.06)	4379 (81.94)		0.48 (0.40–0.56)**
Recent dental visit^c^				
Less than one year	1091 (15.67)	4497 (84.33)	< 0.001	0.43 (0.36–0.50)**
More than one year	1761 (41.12)	2315 (58.88)		1 (ref)
Financial barriers				
Yes	1026 (54.43)	866 (45.57)	< 0.001	2.84 (2.38–3.39)**
No	1832 (19.45)	5949 (80.55)		1 (ref)
Non-financial barriers				
Yes	234 (49.94)	216 (50.06)	< 0.001	1.67 (1.23–2.26)*
No	2624 (23.88)	6599 (76.12)		1 (ref)

The sample size was N = 9673; *CI* Confidence interval

** *p*-value < 0.001; * *p* -value < 0.05

^a^*p*-value from Pearson chi-square statistics comparing the percentage of adults with untreated caries and without untreated caries

^b^Odds ratios were determined by multivariate logistic regression model. The F-adjusted mean residual test for goodness of fit suggests no evidence of lack of fit (*p*-value = 0.236)

^c^Family income categories based on FPL had 746 missing observations; marital status had 301 missing observations; education level had 13 missing observations; recent dental visit had 9 missing observations

The odds of untreated caries among adults ages 19 to 45 years were higher compared to the odds of untreated caries among adults ages 65 and above (OR: 1.47; 95% CI: 1.13–1.91). The odds of untreated caries were lower among other Hispanic adults (OR: 0.72; 95% CI: 0.54–0.95) compared to non-Hispanic White adults. The odds of untreated caries among non-Hispanic Black adults were higher (OR: 1.61; 95% CI: 1.34–1.95) compared to non-Hispanic White adults. Male adults had significantly higher odds of untreated caries compared to the female adults (OR: 1.28; 95% CI: 1.06–1.54). The odds of untreated caries among adults below 100% FPL were 2.4 compared to the odds among adults at 400% FPL and above (OR: 2.40; 95% CI: 1.89–3.05). The odds of untreated caries among adults with some college level education and above were 0.5 compared to those who were not high school graduates (OR: 0.48; 95% CI: 0.40–0.56). Adults who had a recent dental visit within the past year had lower odds of untreated caries (OR: 0.43; 95% CI: 0.36–0.50). The odds of untreated caries among adults with financial barriers were 2.8 (OR: 2.84; 95% CI: 2.38–3.39), whereas the odds of untreated caries among adults with non-financial barriers were only 1.7 (OR: 1.67; 95% CI: 1.23–2.26). Demographic factors such as age, race/ethnicity and gender, socio-economic factors such as family income level and education level, access to care, and utilization of care were significantly associated with untreated caries among adults.

## Discussion

About 70 million U.S. children and adults had untreated caries between 2011 and 2014. Previous studies have noted several socio-economic disparities for dental care among children [10–14]. This study provides further evidence that socioeconomic disparities exist for children. It is possible that the dental coverage mandated for children helps reduce racial/ethnic and gender disparities during childhood, but this trend reverses as children age and coverage disintegrates. Our results show that having a dental visit in the past year is associated with lower odds of untreated caries among children. Frequent or routine dental visits could help prevent tooth decay and other adverse dental conditions while also providing an opportunity for early intervention. This underscores the importance of having access to dental care. We found that all else being equal, the odds of untreated caries associated with non-financial barriers were higher than the odds of untreated caries associated with financial barriers for children. This could be because financial barriers to dental care are lower among children as a result of comprehensive dental coverage through Medicaid and CHIP.

Nonetheless, financial barriers exist for children. Often the cost of dental care is not affordable even for those with dental insurance [22]. Additionally, many plans do not offer first dollar coverage [23] for preventive dental services, which could prevent parents from taking their children in for routine dental care that may prevent caries and may explain the association between family income and untreated caries among children. It is possible that non-financial barriers such as lack of convenient appointment times, parental busyness, prohibitive distance to dentist’s office, or a child’s fear of the dentist [24] play a critical role in untreated caries among children and deserve more attention. Interventions such as school-based sealant programs may help address non-financial barriers by bringing the dentist to children and eliminating travel and time-related barriers [25, 26].

Our study supports findings in previous studies on socio-economic and racial disparities among adults. [8, 9] An early study of the impact of Medicaid expansion on adult oral health showed a small increase in dental care utilization among adults in states that expanded Medicaid [27]. While this study did not address untreated caries specifically, the expansion of Medicaid and inclusion of extensive dental benefits for adults could reduce socio-economic disparities among low-income adults. Similar to children, our results show that having a dental visit in the past year is associated with lower odds of untreated caries among adults. Utilization of dental care can help to mitigate tooth decay and other dental conditions.

The percentage of adults with financial barriers to dental care who had untreated caries (54.4%) was close to the percentage of adults with non-financial barriers to dental care who had untreated caries (49.9%). This suggests that access to dental care when needed is a significant issue for adults and is not strictly financial. Rather, there are myriad obstacles to obtaining dental care for U.S. adults, suggesting that a larger policy change may be necessary to prevent caries or to obtain treatment for caries. Still, all else being equal, the odds of untreated caries associated with financial barriers for adults are higher than the odds of untreated caries associated with non-financial barriers. In other words, addressing financial barriers to dental care among adults is likely to lead to a larger reduction in untreated caries than addressing non-financial barriers. This result differs from what we see for children and could be explained by the fact that dental insurance coverage rates for adults are much lower than for children. In 2014, 29.4% of adults ages 19 to 64 and 62.0% of adults ages 65 and above had no form of dental insurance whereas only 11.0% of children ages 2 to 18 had no form of dental insurance [5]. In fact, adults report a higher rate of financial barriers to dental care than for any other health care service, including prescription drugs and eyeglasses [6]. While non-financial barriers also need to be addressed, making dental care more affordable could help to reduce untreated caries among adults. The out-of-pocket spending for dental services on average is about $310 per adult irrespective of their insurance status [28]. The total expenditure for dental care for non-elderly adults under 100% FPL is about $523 per adult [28]. This expense may be cost prohibitive for the majority of adults with lower family incomes and may explain why they have higher odds of untreated caries.

In our study, we also observed that the odds of untreated caries among adults ages 19–45 years and 46–64 years were higher as compared to those among adults ages 65 years and above. This may be because adults over the age of 65 only have 18.9 remaining teeth, on average [29]. Since we do not include missing teeth in our measure of untreated caries, it is possible that the odds of decay are higher among adults under 65 years as compared to adults 65 years and above simply because younger adults have more opportunity to experience decay.

Further, higher education levels were also associated with lower odds of untreated caries among adults. Education level is a predictor of health outcomes and influences health on individual, community, and social levels [30]. It is possible that adults with higher levels of education have greater awareness of self-health, health literacy, access to resources, and understanding of how to navigate the health care system. One study has also noted the association of higher education level with better oral health status [31].

Moving forward, policymakers ought to explore ways to ease dental care affordability challenges for children and adults, particularly those that are low income, such as including comprehensive dental coverage for adults within Medicaid and Medicare. A previous study shows that cost barriers to dental care decreased for low-income adults in part due to Medicaid expansion [6]. Recent analysis estimates that it would cost state Medicaid programs, on average, an additional 1.5% of current state Medicaid spending to provide extensive adult dental benefits within Medicaid [32]. This does not include the fiscal offsets that are likely to accrue such as reduced hospital emergency room spending and reduced medical care costs among Medicaid beneficiaries with chronic diseases. In addition, expanding Medicare to cover dental services may also improve affordability for seniors and thus improve accessibility of dental care services.

Innovative use of technology such as teledentistry may also help to reduce untreated caries. Teledentistry is a valid and cost-effective tool for screening dental caries [33, 34]. Use of teledentistry for dental interventions led to improved clinical outcomes [33]. Teledentistry could help to reduce non-financial barriers such as travel time [35] and improve access to dental care among underserved populations [36]. Further, an integration of medicine and dentistry [37] could also help improve the access and delivery of oral health services by collocating oral health services with medical services that individuals are more likely to use.

Finally, water fluoridation plays an important part in preventing and reducing tooth decay by 25% [38]. However, only 74.4% of the U.S. population live on community water systems that receive fluoridated water. Hawaii, Oregon and New Jersey have less than 25% of their population on community water systems that receive fluoridated water. [39] Brushing teeth using a fluoridated toothpaste can also help to protect teeth and prevent decay [40]. Promoting water fluoridation and fluoridated toothpaste use could help to prevent and reduce tooth decay throughout the United States.

There are a few limitations to this study. In particular, this study did not account for the type of insurance the participants had. The NHANES data on health insurance does not specify if surveyed individuals had dental insurance and hence was not considered in this study. Furthermore, this study did not account for rural-urban differences due to lack of data availability. This study focused only on the presence of untreated caries and not the severity of untreated caries. This study is cross-sectional, so it can only establish associations. It cannot establish causality.

## Conclusions

About one sixth of the child population and about one quarter of the adult population in the U.S. had untreated caries from 2011 to 2014. There are differences in the disparities for untreated caries among children and adults. Age, family income level, recent dental visit, and financial and non-financial barriers were significantly associated with untreated caries in both children and adults. Race/ethnicity, gender and education level were also significantly associated with untreated caries among adults.

## Abbreviations

ACA: Affordable care act
CHIP: Children’s Health Insurance Program
FPL: Federal poverty level
NHANES: National Health and Nutrition Examination Survey
